# Supplementary material for: The Systems Biology Research Tool: evolvable open-source software
Source: BMC Syst Biol. 2008 Jun 29;2:55. doi: 10.1186/1752-0509-2-55 (PMC2446383; doi:10.1186/1752-0509-2-55)
Supplement: Additional file 1 — SBRT Archive. An archive of the current version of the Systems Biology Research Tool. [file 1752-0509-2-55-S1.zip › sbrt-1.4.0/doc/developers_guide/api/sbrt/kernel/math/class-use/MathExpr.html]

Uses of Interface sbrt.kernel.math.MathExpr


|  |  |  |
| --- | --- | --- |
| **Overview** \| **Package** \| **Class** \| **Use** \| **Tree** \| **Deprecated** \| **Index** \| **Help** | |  |
| PREV   NEXT | **FRAMES**    **NO FRAMES**     **All Classes** |  |


---


## **Uses of Interface sbrt.kernel.math.MathExpr**

| Packages that use MathExpr | |
| --- | --- |
| **sbrt.ext.cplex** | Provides a class that allows communication with CPLEX. |
| **sbrt.ext.glpk** | Provides a class that allows communication with GLPK. |
| **sbrt.kernel.fba** | Provides classes and interfaces for creating objects relevant to Flux Balance Analysis. |
| **sbrt.kernel.fba.comp** | Provides classes and interfaces for performing Flux Balance Analysis computations. |
| **sbrt.kernel.fba.proc** | Provides classes and interfaces for executing Flux Balance Analysis processes. |
| **sbrt.kernel.math** | Provides classes and interfaces for creating objects relevant to mathematics. |
| **sbrt.kernel.math.optimization** | Provides classes and interfaces for creating objects relevant to solving optimization problems. |
| **sbrt.kernel.utilities.proc** | Provides classes and interfaces for performing utility processes. |
| **sbrt.shell.io.fba** | Provides classes and interfaces for reading and writing files relevant to Flux Balance Analysis. |
| **sbrt.shell.text.fba** | Provides classes and interfaces for defining formats for objects relevant to Flux Balance Analysis. |
| **sbrt.shell.text.math** | Provides classes and interfaces for defining formats for mathematical objects. |

Uses of MathExpr in sbrt.ext.cplex

| Methods in sbrt.ext.cplex with parameters of type MathExpr | |
| --- | --- |
| `void` | `CplexSolver.setConstraint(MathExpr<? extends C> expression, Interval c)`             Sets a constraint on the provided mathematical expression of column variables. |
| `void` | `CplexSolver.setObjectiveFunction(MathExpr<? extends C> objective)`             Sets the objective function to the provided mathematical expression of column variables. |
| `void` | `CplexSolver.setObjectiveFunction(MathExpr<? extends C> objective, OptimizationSense sense)`             Sets the objective function and optimization sense to the provided values. |

| Method parameters in sbrt.ext.cplex with type arguments of type MathExpr | |
| --- | --- |
| `void` | `CplexSolver.setConstraints(java.util.Map<? extends MathExpr<? extends C>,Interval> exprConstraints)`             Sets constraints on the provided mathematical expressions of column variables. |

Uses of MathExpr in sbrt.ext.glpk

| Methods in sbrt.ext.glpk with parameters of type MathExpr | |
| --- | --- |
| `void` | `GlpkSolver.setConstraint(MathExpr<? extends C> expression, Interval c)`             Sets a constraint on the provided mathematical expression of column variables. |
| `void` | `GlpkSolver.setObjectiveFunction(MathExpr<? extends C> expression)`             Sets the objective function to the provided mathematical expression of column variables. |
| `void` | `GlpkSolver.setObjectiveFunction(MathExpr<? extends C> expression, OptimizationSense sense)`             Sets the objective function and optimization sense to the provided values. |

| Method parameters in sbrt.ext.glpk with type arguments of type MathExpr | |
| --- | --- |
| `void` | `GlpkSolver.setConstraints(java.util.Map<? extends MathExpr<? extends C>,Interval> expressionConstraints)`             Sets constraints on the provided mathematical expressions of column variables. |

Uses of MathExpr in sbrt.kernel.fba

| Methods in sbrt.kernel.fba that return types with arguments of type MathExpr | |
| --- | --- |
| `UnmodifiableMap<MathExpr<java.lang.String>,Interval>` | `FluxConstraints.getExprConstraints()`             Returns the constraints for mathematical expressions of fluxes. |

| Methods in sbrt.kernel.fba with parameters of type MathExpr | |
| --- | --- |
| `Interval` | `FluxConstraints.getConstraint(MathExpr<java.lang.String> rxnExpr)`             Returns the constraint of the provided mathematical expression of reaction names. |
| `Interval` | `Fluxome.getRxnExprConstraint(MathExpr<java.lang.String> rxnNameExpr)`             Returns the constraint corresponding to the provided mathematical expression of reaction names. |
| `Interval` | `IrrevRxnFluxomeV1.getRxnExprConstraint(MathExpr<java.lang.String> rxnNameExpr)`             Returns the constraint associated with the expression provided. |

Constructor parameters in sbrt.kernel.fba with type arguments of type MathExpr

`FluxConstraints(java.util.Map<java.lang.String,Interval> fluxConstraints,
java.util.Map<? extends MathExpr<java.lang.String>,Interval> exprConstraints)`
  
          Constructs a set of FBA constraints from the provided arguments.

Uses of MathExpr in sbrt.kernel.fba.comp

| Methods in sbrt.kernel.fba.comp with type parameters of type MathExpr | |
| --- | --- |
| `<V,E extends MathExpr<V>> java.util.Map<E,java.lang.Double>` | `FbaOptComputer.getMaxima(java.util.Collection<E> objectives)`             Computes the maximum value of each provided objective function. |
| `<V,E extends MathExpr<V>> java.util.Map<E,java.lang.Double>` | `FbaOptComputer.getMinima(java.util.Collection<E> objectives)`             Computes the minimum value of each provided objective function. |

| Methods in sbrt.kernel.fba.comp that return MathExpr | |
| --- | --- |
| `MathExpr` | `FluxCapException.getFluxCap()`             Returns the invalid flux cap. |
| `MathExpr<java.lang.String>` | `RxnDeletionComputer.getObjectiveFunction()`             Returns the current objective function. |
| `MathExpr<?>` | `FbaOptException.getObjFunction()`             Returns the objective function whose optimal value could not be found. |

| Methods in sbrt.kernel.fba.comp that return types with arguments of type MathExpr | |
| --- | --- |
| `java.util.Map<MathExpr<?>,Interval>` | `FbaOptComputer.getColExprConstraints()`             Returns all current constraints on mathematical expressions of column variables. |
| `java.util.Set<MathExpr<?>>` | `FbaOptComputer.getColExprs()`             Returns the set of all mathematical expressions of column variables. |
| `java.util.Map<MathExpr<java.lang.String>,Interval>` | `RxnDeletionComputer.getRxnExprConstraints()`             Returns the set of all constraints on mathematical expressions of fluxes. |
| `java.util.Map<MathExpr<java.lang.String>,Interval>` | `InitialPointComputer.getRxnExprConstraints()`             Returns all current flux expression constraints. |
| `java.util.Map<? extends MathExpr<java.lang.String>,Interval>` | `FluxCvComputer.getRxnExprConstraints()`             Returns all current flux expression constraints. |
| `java.util.Map<MathExpr<java.lang.String>,Interval>` | `AbstractFluxIntervalComputer.getRxnExprConstraints()`             Returns all current flux expression constraints. |
| `java.util.Map<MathExpr<java.lang.String>,Interval>` | `FbaOptComputer.getRxnExprConstraints()`             Returns all current constraints on mathematical expressions of reaction names. |
| `java.util.Map<MathExpr<java.lang.String>,Interval>` | `FastMsFluxRangeComputer.getRxnExprConstraints()`             Returns all current flux expression constraints. |
| `java.util.Set<MathExpr<java.lang.String>>` | `FbaOptComputer.getRxnExprs()`             Returns the set of all mathematical expressions of reaction names. |

| Methods in sbrt.kernel.fba.comp with parameters of type MathExpr | |
| --- | --- |
| `protected  void` | `FbaOptComputer.checkColExpr(MathExpr<?> e)`             Ensures all the variables in the provided expression are valid column variables. |
| `protected  void` | `AbstractCvComputer.checkRxnExpr(MathExpr<java.lang.String> e)`             Ensures the expression is valid for the fluxome provided. |
| `protected  void` | `FbaOptComputer.checkRxnExpr(MathExpr<java.lang.String> e)`             Ensures all the variables in the provided expression are reaction names. |
| `protected  double` | `AbstractFluxIntervalComputer.getBoundedMax(MathExpr<java.lang.String> rxnNameExpr)`             Computes the maximum value of the provided objective function. |
| `protected  double` | `AbstractFluxIntervalComputer.getBoundedMin(MathExpr<java.lang.String> rxnNameExpr)`             Computes the minimum value of the provided objective function. |
| `Interval` | `FbaOptComputer.getColExprConstraint(MathExpr<?> columnVariableExpr)`             Returns the current constraint on the provided expression. |
| `FbaOptComputer.Results` | `FbaOptComputer.getMax(MathExpr<?> objective)`             Returns the results of computing the maximum value of the provided objective function. |
| `double` | `FbaOptComputer.getMaximum(MathExpr<?> objective)`             Computes the maximum value of the provided objective function. |
| `protected  double` | `AbstractFluxIntervalComputer.getMaximum(MathExpr<java.lang.String> rxnNameExpr)`             Computes the maximum value of the provided objective function. |
| `FbaOptComputer.Results` | `FbaOptComputer.getMin(MathExpr<?> objective)`             Returns the results of computing the minimum value of the provided objective function. |
| `double` | `FbaOptComputer.getMinimum(MathExpr<?> objective)`             Computes the minimum value of the provided objective function. |
| `protected  double` | `AbstractFluxIntervalComputer.getMinimum(MathExpr<java.lang.String> rxnNameExpr)`             Computes the minimum value of the provided objective function. |
| `FbaOptComputer.Results` | `FbaOptComputer.getOptimal(MathExpr<?> objective, OptimizationSense sense)`             Returns the results of computing the optimal value of the provided objective function. |
| `Interval` | `RxnDeletionComputer.getRxnExprConstraint(MathExpr<java.lang.String> rxnNameExp)`             Returns the constraint on the provided mathematical expression of fluxes. |
| `Interval` | `InitialPointComputer.getRxnExprConstraint(MathExpr<java.lang.String> rxnNameExp)`             Returns the current constraint on the provided expression. |
| `Interval` | `FluxCvComputer.getRxnExprConstraint(MathExpr<java.lang.String> rxnNameExp)`             Returns the current constraint on the provided expression. |
| `Interval` | `CdHarFluxVectorComputer.getRxnExprConstraint(MathExpr<java.lang.String> rxnNameExp)`             Returns the current constraint on the provided expression. |
| `Interval` | `AbstractFluxIntervalComputer.getRxnExprConstraint(MathExpr<java.lang.String> rxnNameExp)`             Returns the current constraint on the provided expression. |
| `Interval` | `FbaOptComputer.getRxnExprConstraint(MathExpr<java.lang.String> rxnNameExpr)`             Returns the current constraint on the provided expression. |
| `Interval` | `FastMsFluxRangeComputer.getRxnExprConstraint(MathExpr<java.lang.String> rxnNameExp)`             Returns the current constraint on the provided expression. |
| `void` | `FbaOptComputer.restoreColExprConstraint(MathExpr<?> columnVarExpr)`             Restores the constraint on the provided expression of column variables to the value contained in the fluxome. |
| `void` | `RxnDeletionComputer.restoreRxnExprConstraint(MathExpr<java.lang.String> rxnNameExp)`             Restores the constraint on the provided flux expression to the value contained in the fluxome. |
| `void` | `InitialPointComputer.restoreRxnExprConstraint(MathExpr<java.lang.String> expr)`             Restores the constraint on the provided flux expression to the value contained in the fluxome. |
| `void` | `FluxCvComputer.restoreRxnExprConstraint(MathExpr<java.lang.String> rxnNameExp)`             Restores the constraint on the provided flux expression to the value contained in the fluxome. |
| `void` | `CdHarFluxVectorComputer.restoreRxnExprConstraint(MathExpr<java.lang.String> expr)`             Restores the constraint on the provided flux expression to the value contained in the fluxome. |
| `void` | `AbstractFluxIntervalComputer.restoreRxnExprConstraint(MathExpr<java.lang.String> rxnNameExp)`             Restores the constraint on the provided flux expression to the value contained in the fluxome. |
| `void` | `FbaOptComputer.restoreRxnExprConstraint(MathExpr<java.lang.String> rxnNameExpr)`             Restores the constraint on the provided flux expression to the value contained in the fluxome. |
| `void` | `FastMsFluxRangeComputer.restoreRxnExprConstraint(MathExpr<java.lang.String> rxnNameExpr)`             Restores the constraint on the provided flux expression to the value contained in the fluxome. |
| `void` | `FbaOptComputer.setColExprConstraint(MathExpr<?> expr, Interval c)`             Changes the constraint on the provided mathematical expression of column variables. |
| `void` | `RxnDeletionComputer.setObjectiveFunction(MathExpr<java.lang.String> objective)`             Sets the objective function to the provided mathematical expression. |
| `void` | `RxnDeletionComputer.setRxnExprConstraint(MathExpr<java.lang.String> e, Interval c)`             Changes the constraint on the provided mathematical expression of fluxes. |
| `void` | `InitialPointComputer.setRxnExprConstraint(MathExpr<java.lang.String> e, Interval c)`             Changes the constraint on the provided mathematical expression of fluxes. |
| `void` | `FluxCvComputer.setRxnExprConstraint(MathExpr<java.lang.String> e, Interval c)`             Changes the constraint on the provided mathematical expression of fluxes. |
| `void` | `CdHarFluxVectorComputer.setRxnExprConstraint(MathExpr<java.lang.String> rxnExpr, Interval c)`             Changes the constraint on the provided mathematical expression of fluxes. |
| `void` | `AbstractFluxIntervalComputer.setRxnExprConstraint(MathExpr<java.lang.String> e, Interval c)`             Changes the constraint on the provided mathematical expression of fluxes. |
| `void` | `FbaOptComputer.setRxnExprConstraint(MathExpr<java.lang.String> expr, Interval c)`             Changes the constraint on the provided mathematical expression of fluxes. |
| `void` | `FastMsFluxRangeComputer.setRxnExprConstraint(MathExpr<java.lang.String> e, Interval c)`             Changes the constraint on the provided mathematical expression of fluxes. |

| Method parameters in sbrt.kernel.fba.comp with type arguments of type MathExpr | |
| --- | --- |
| `void` | `FbaOptComputer.restoreColExprConstraints(java.util.Collection<? extends MathExpr<?>> colVarExprs)`             Restores the constraints for the provided column variable expressions to those in the provided fluxome. |
| `void` | `InitialPointComputer.restoreRxnExprConstraints(java.util.Collection<? extends MathExpr<java.lang.String>> rxnNameExps)`             Restores the constraints on the provided expressions to the values contained in the fluxome. |
| `void` | `FluxCvComputer.restoreRxnExprConstraints(java.util.Collection<? extends MathExpr<java.lang.String>> rxnNameExps)`             Restores the constraints on the provided expressions to the values contained in the fluxome. |
| `void` | `AbstractCvComputer.restoreRxnExprConstraints(java.util.Collection<? extends MathExpr<java.lang.String>> rxnNameExps)`             Restores the constraints on the provided expressions to the values contained in the fluxome. |
| `void` | `AbstractFluxIntervalComputer.restoreRxnExprConstraints(java.util.Collection<? extends MathExpr<java.lang.String>> rxnNameExps)`             Restores the constraints on the provided expressions to the values contained in the fluxome. |
| `void` | `FbaOptComputer.restoreRxnExprConstraints(java.util.Collection<? extends MathExpr<java.lang.String>> rxnNameExprs)`             Restores the constraints for the provided reaction name expressions to those in the provided fluxome. |
| `void` | `FastMsFluxRangeComputer.restoreRxnExprConstraints(java.util.Collection<? extends MathExpr<java.lang.String>> rxnNameExprs)`             Restores the constraints on the provided expressions to the values contained in the fluxome. |
| `void` | `FbaOptComputer.setColExprConstraints(java.util.Map<? extends MathExpr<?>,Interval> exprConstraints)`             Changes the constraints on more than one mathematical expression of column variables. |
| `void` | `InitialPointComputer.setRxnExprConstraints(java.util.Map<? extends MathExpr<java.lang.String>,Interval> constraints)`             Changes the constraints on more than one mathematical expression of fluxes. |
| `void` | `FluxCvComputer.setRxnExprConstraints(java.util.Map<? extends MathExpr<java.lang.String>,Interval> constraints)`             Changes the constraints on more than one mathematical expression of fluxes. |
| `void` | `AbstractCvComputer.setRxnExprConstraints(java.util.Map<? extends MathExpr<java.lang.String>,Interval> constraints)`             Changes the constraints on more than one mathematical expression of fluxes. |
| `void` | `AbstractFluxIntervalComputer.setRxnExprConstraints(java.util.Map<? extends MathExpr<java.lang.String>,Interval> constraints)`             Changes the constraints on more than one mathematical expression of fluxes. |
| `void` | `FbaOptComputer.setRxnExprConstraints(java.util.Map<? extends MathExpr<java.lang.String>,Interval> exprConstraints)`             Changes the constraints on more than one mathematical expression of fluxes. |
| `void` | `FastMsFluxRangeComputer.setRxnExprConstraints(java.util.Map<? extends MathExpr<java.lang.String>,Interval> constraints)`             Changes the constraints on more than one mathematical expression of fluxes. |

Constructors in sbrt.kernel.fba.comp with parameters of type MathExpr

`FbaOptException(MathExpr<?> objectiveFunction,
OptimizationSense sense,
ProgramSolverStatus status)`
  
          Constructs a new FBA optimization exception.

`FbaOptException(MathExpr<?> objectiveFunction,
OptimizationSense sense,
ProgramSolverStatus status,
java.lang.String message)`
  
          Constructs a new FBA optimization exception.

`FluxCapException(MathExpr fluxCap,
java.lang.String reactionName)`
  
          Constructs a new flux cap exception.

`FluxCapException(MathExpr fluxCap,
java.lang.String reactionName,
java.lang.Throwable cause)`
  
          Constructs a new flux cap exception.

Uses of MathExpr in sbrt.kernel.fba.proc

| Methods in sbrt.kernel.fba.proc that return MathExpr | |
| --- | --- |
| `MathExpr<java.lang.String>` | `CvFbaOptimizationExperiment.getObjectiveFunction()`             Returns the current objective function. |
| `MathExpr<java.lang.String>` | `CatalystDeletionProcess.getObjectiveFunction()`             Returns the current objective function. |
| `MathExpr<java.lang.String>` | `RxnDeletionProcess.getObjectiveFunction()`             Returns the current objective function. |
| `MathExpr<java.lang.String>` | `CvRxnDeletionProcess.getObjectiveFunction()`             Returns the current objective function. |
| `MathExpr<java.lang.String>` | `StoichVariationProcess.getObjectiveFunction()`             Returns the current objective function. |
| `MathExpr<java.lang.String>` | `CvCatalystDeletionProcess.getObjectiveFunction()`             Returns the current objective function. |

| Methods in sbrt.kernel.fba.proc with parameters of type MathExpr | |
| --- | --- |
| `void` | `CvFbaOptimizationExperiment.setObjectiveFunction(MathExpr<java.lang.String> objective)`             Sets the objective function to the provided mathematical expression. |
| `void` | `CatalystDeletionProcess.setObjectiveFunction(MathExpr<java.lang.String> objective)`             Sets the objective function to the provided mathematical expression. |
| `void` | `RxnDeletionProcess.setObjectiveFunction(MathExpr<java.lang.String> objective)`             Sets the objective function to the provided mathematical expression. |
| `void` | `CvRxnDeletionProcess.setObjectiveFunction(MathExpr<java.lang.String> objective)`             Sets the objective function to the provided mathematical expression. |
| `void` | `StoichVariationProcess.setObjectiveFunction(MathExpr<java.lang.String> objective)`             Sets the objective function to the provided mathematical expression. |
| `void` | `CvCatalystDeletionProcess.setObjectiveFunction(MathExpr<java.lang.String> objective)`             Sets the objective function to the provided mathematical expression. |

| Method parameters in sbrt.kernel.fba.proc with type arguments of type MathExpr | |
| --- | --- |
| `void` | `ObjectiveFunctionAnalysis.setObjectiveFunctionSource(java.lang.Iterable<? extends MathExpr<java.lang.String>> objectiveSource)`             Sets the objective function input source. |
| `void` | `CvObjectiveFunctionProcess.setObjectiveFunctionSource(java.lang.Iterable<? extends MathExpr<java.lang.String>> objectiveSource)`             Sets the objective function input source. |

Uses of MathExpr in sbrt.kernel.math

| Subinterfaces of MathExpr in sbrt.kernel.math | |
| --- | --- |
| `interface` | `LinearComb<V>`             This interface is used to represent linear combinations. |
| `interface` | `Polynomial<V>`             This interface is used to represent polynomials. |
| `interface` | `QuadraticExpr<V>`             This interface is used to represent quadratic expressions. |

| Classes in sbrt.kernel.math that implement MathExpr | |
| --- | --- |
| `class` | `AbstractLinearComb<V>`             This abstract class is a skeleton implementation of `LinearComb`. |
| `class` | `AbstractPolynomial<V>`             This abstract class is a skeleton implementation of `Polynomial`. |
| `class` | `HashLinearComb<V>`             This class is a concrete implemenation of `LinearComb`. |

| Methods in sbrt.kernel.math that return MathExpr | |
| --- | --- |
| `MathExpr<V>` | `MathExpr.plugInNumbers(java.util.Map<?,java.lang.Double> variableValues)`             Plugs in the provided numerical values to produce a new mathematical expression. |

Uses of MathExpr in sbrt.kernel.math.optimization

| Methods in sbrt.kernel.math.optimization with parameters of type MathExpr | |
| --- | --- |
| `void` | `ProgramSolver.setConstraint(MathExpr<? extends C> expression, Interval c)`             Sets a constraint on the provided mathematical expression of column variables. |
| `void` | `ProgramSolver.setObjectiveFunction(MathExpr<? extends C> expression)`             Sets the objective function to the provided mathematical expression of column variables. |
| `void` | `ProgramSolver.setObjectiveFunction(MathExpr<? extends C> expression, OptimizationSense sense)`             Sets the objective function and optimization sense to the provided values. |

| Method parameters in sbrt.kernel.math.optimization with type arguments of type MathExpr | |
| --- | --- |
| `void` | `ProgramSolver.setConstraints(java.util.Map<? extends MathExpr<? extends C>,Interval> expressionConstraints)`             Sets constraints on the provided mathematical expressions of column variables. |

Uses of MathExpr in sbrt.kernel.utilities.proc

| Classes in sbrt.kernel.utilities.proc with type parameters of type MathExpr | |
| --- | --- |
| `class` | `VariableParticipation<E extends MathExpr<V>,V>`             This class is used to organize a collection of mathematical expressions according to the variables they contain. |

Uses of MathExpr in sbrt.shell.io.fba

| Classes in sbrt.shell.io.fba with type parameters of type MathExpr | |
| --- | --- |
| `class` | `RxnNamExprSetInputFile<E extends MathExpr<S>,S>`             This class is used to read files containing a set of reaction name expressions. |

Uses of MathExpr in sbrt.shell.text.fba

| Classes in sbrt.shell.text.fba with type parameters of type MathExpr | |
| --- | --- |
| `interface` | `RxnNameExprFormat<E extends MathExpr<S>,S>`             This interface is used to represent formats for mathematical expressions of reaction names. |

Uses of MathExpr in sbrt.shell.text.math

| Classes in sbrt.shell.text.math with type parameters of type MathExpr | |
| --- | --- |
| `interface` | `MathExprFormat<E extends MathExpr<V>,V>`             This interface is used to represent formats for linear combinations. |

---


|  |  |  |
| --- | --- | --- |
| **Overview** \| **Package** \| **Class** \| **Use** \| **Tree** \| **Deprecated** \| **Index** \| **Help** | |  |
| PREV   NEXT | **FRAMES**    **NO FRAMES**     **All Classes** |  |


---
